# The genetic variation of *Angiostrongylus cantonensis* in the People’s Republic of China

**DOI:** 10.1186/s40249-017-0341-z

**Published:** 2017-09-01

**Authors:** Shan Lv, Yi Zhang, Peter Steinmann, Jürg Utzinger, Xiao-Nong Zhou

**Affiliations:** 1 National Institute of Parasitic Diseases, Chinese Center for Disease Control and Prevention, Shanghai, 200025 People’s Republic of China; 2 Swiss Tropical and Public Health Institute, P.O. Box, CH-4002 Basel, Switzerland; 3 University of Basel, P.O. Box, CH-4003 Basel, Switzerland

**Keywords:** *Angiostrongylus cantonensis*, Genetics, *cox*1, *nad*1, Internal transcribed spacer, People’s Republic of China

## Abstract

**Background:**

The People’s Republic of China (P.R. China) is the presumptive home range of the rat lungworm *Angiostrongylus cantonensis*, a major aetiological agent of human eosinophilic meningitis. We present a study of the genetic variation of *A. cantonensis* in P.R. China. Our aim was to deepen the current knowledge pertaining to its origin and global spread from a molecular perspective.

**Methods:**

Adult *A. cantonensis* were collected in the frame of a national survey and identified based on morphological criteria. Polymerase chain reaction (PCR) was employed to amplify the target DNA sequences (cytochrome c oxidase subunit I (*cox1*), nicotinamide adenine dinucleotide dehydrogenase subunit 1 (*nad1*) and internal transcribed spacer (ITS)). The PCR product of *cox*1 was directly submitted to sequencing, while clone sequencing was used for *nad*1 and ITS. The identity of the samples was verified by comparing the sequences to those of accepted *A. cantonensis* specimens. The specific composition of substitutions in each gene was analysed, and the genotypes were compared based on the complete *cox*1, *nad*1 and ITS genes.

**Results:**

We characterised the complete mitochondrial genes *cox*1 and *nad*1 of 130 specimens and obtained 357 nuclear sequences containing two complete ITS (ITS1 and ITS2) and 5.8S rRNA of the same samples. All specimens were genetically confirmed as *A. cantonensis*. Two major groups (i.e. I and II) were identified according to the phylogeny of *cox*1 sequences. Group I could be further categorised into six distinct clades. Almost half of the specimens (47.7%) belong to the clade Ia and 22.3% to the group II. The former was widely distributed across the study region. A variable number of repeat units in three microsatellites was observed, resulting in considerable length variation in ITS. Intragenomic variation of ITS sequences was found in a large proportion of the samples. Genotyping showed a striking difference between mitochondrial DNA and ITS.

**Conclusions:**

Our results demonstrate that *A. cantonensis* is the only rat lungworm species in P.R. China and shows high genetic diversity. Results of diversity and genotyping of *A. cantonensis* can be impacted by the sequencing strategy and biomarker. Although ITS may be a valuable marker for interspecific identification, it is not suitable for studying the intraspecific variation of *A. cantonensis* due to its high intragenomic variation and current challenges for direct sequencing.

**Electronic supplementary material:**

The online version of this article (doi:10.1186/s40249-017-0341-z) contains supplementary material, which is available to authorized users.

## Multilingual abstracts

Please see Additional file 1 for translations of the abstract into the six official working languages of the United Nations.

## Background

The rat lungworm *Angiostrongylus cantonensis* is an important aetiological agent of human eosinophilic meningitis [1]. The life cycle of *A. cantonensis* involves rats as definitive host and mollusks as intermediate host. Humans acquire infections mainly through the consumption of undercooked snails that harbour infective larvae. Humans are not permissive hosts. Larvae mainly dwell in the vessels of the central nervous system (CNS) and only rarely migrate to the pulmonary arteries [2]. Inflammation in the CNS is the proximate cause of morbidity due to an infection with *A. cantonensis*. Severe headache and paresthesia are the most common symptoms. Light infections are usually self-limiting, but heavy infections can be fatal, with young children at highest risk [3]. Globally, more than 3000 human cases have been reported [1].

There is consensus that *A. cantonensis* originated from the southern regions of the People’s Republic of China (P.R. China) [4] or the Indochinese peninsula [5]. In the wake of the global spread of rats (*Rattus rattus* and *R. norvegicus*) and certain snail species (e.g. *Achatina fulica* and *Pomacea* spp.) [6], *A. cantonensis* is spreading across the tropics and subtropics. Thus far, over 30 countries have reported local *A. cantonensis* transmission [1], and recent reports suggest a further expansion [7–12].

Molecular evidence can deepen the understanding of the global spread of *A. cantonensis*. Two molecular markers (i.e. cytochrome c oxidase subunit I (*cox*1) and internal transcribed spacer (ITS)), have been employed to elucidate the phylogeny of *A. cantonensis* [7, 13–18]. In general, *cox*1 proved to be a particularly useful marker for phylogenetic inference, whereas ITS is more valuable for interspecies differentiation.

*A. cantonensis* was first reported from Guangzhou, P.R. China, in 1935 [19]. A probably identical nematode was described in Taiwan two years later [20]. These early observations indicate that the South of P.R. China and offshore islands constituted the native range of *A. cantonensis*. However, the full extent of the endemic area was not revealed until recently [3, 21] and the genetic diversity of *A. cantonensis* remains to be fully elucidated [14, 18, 22]. Insights into the genetic diversity of *A. cantonensis* in P.R. China will contribute to the existing knowledge and be helpful to clarify the global spread of the parasite. The aim of this study was to present the genetic variation of *A. cantonensis* in P.R. China to deepen the current knowledge-base pertaining to its origin and global spread from a molecular perspective.

## Methods

### Collection of *A. cantonensis* specimens and total DNA preparation

**Fig. 1:**
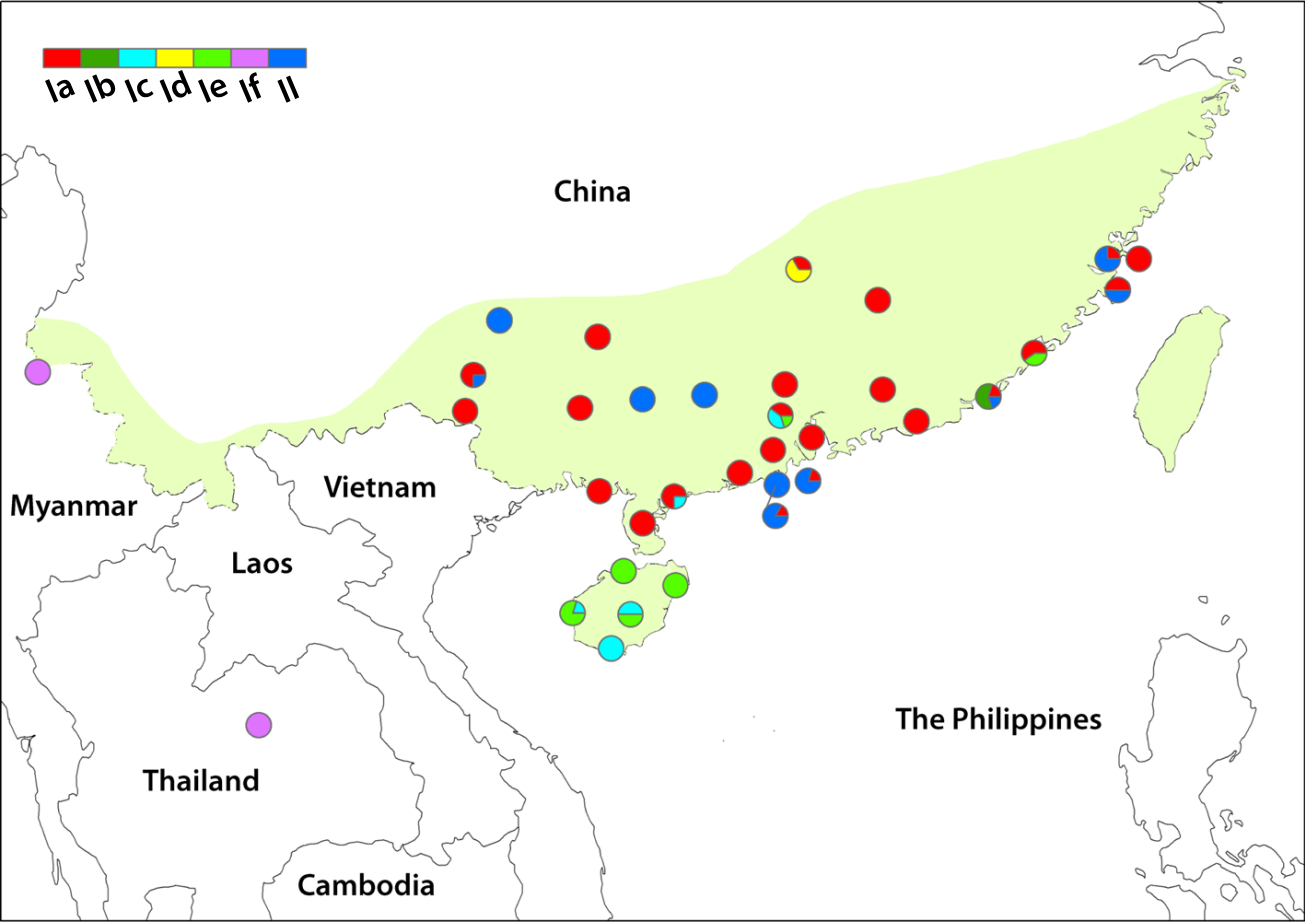
Geographical distribution of *cox*1 clades of *Angiostrongylus cantonensis* in P.R. China. Two groups, i.e. I and II, are shown. Six clades are further distinguished in group I (Ia, Ib, Ic, Id, Ie and If). The current endemic area of *A. cantonensis* in P.R. China is indicated by shading

*A. cantonensis* specimens were collected in the frame of the first national survey of the distribution of angiostrongyliasis in the mainland of P.R. China, which was implemented in 2006 and 2007 [21] (Fig. 1). Wild rats (*R. norvegicus*, *R. rattus* and *R. flavipectus*) and mollusks were collected and examined for the presence of *Angiostrongylus* spp., based on morphological criteria of adult worms. In localities where adult worms could not be discovered in rats, the larvae were collected from infected *Pomacea* spp. and/or *Achatina fulica* snails. Sprague-Dawley (SD) rats were infected with pools of 20–50 larvae in laboratory. Adult worms were then collected from the rats six weeks post-infection and identified morphologically. All adult *Angiostrongylus* worms were kept in 75% ethanol pending further genetic identification. For comparison, *A. cantonensis* DNA specimens from Thailand were kindly provided by Dr. Praphathip Eamsobhana from Mahidol University, using a Flinders Technology Associates (FTA) card [16].

*A. cantonensis* specimens were individually washed three times using phosphate buffered saline (PBS) and placed into clean 1.5 ml tubes. Worms were then cut into small pieces and incubated with sodium dodecyl-sulphate/proteinase K at 56 °C, pH 7.4, for 4–6 h [23]. The suspension was centrifuged and the supernatant transferred into another tube for extraction with phenol/chloroform/isoamyl alcohol (v:v:v = 25:24:1). The DNA pellet was suspended in 30–50 μl H_2_O and kept at −20 °C pending analysis.

### Polymerase chain reaction (PCR) and sequencing

The primers targeting two mitochondrial genes, *nad*1 and *cox*1, were designed according to the complete mitochondrial genome of *A. cantonensis* (GQ398121) (Table 1). To obtain the full gene sequence of *cox*1, two overlapping fragments were amplified. We employed universal primers for the complete sequence of two nuclear ITS (i.e. ITS1 and ITS2) and 5.8S ribosomal RNA genes [18]. PCR was performed in 50 μl with 1.5 mM of MgCl_2_, 10 μM of each primer, 25 μl 2 × *Taq* buffer, 0.2 mM of each dNTPs, 2.5 U of *Taq* DNA polymerase and 1 μl of DNA sample as follows: 94 °C for 5 min, 35 cycles at 94 °C for 60 s, around 48–55 °C for 60 s, and 72 °C for 60–90 s, followed by 72 °C for 10 min for the final extension.

**Table 1:** Primers used in the present study to determine the genetic structure of *A. cantonensis* in P.R. China

Gene	Primer	Primer sequence	Annealing temperature
*nad*1	ND1_F	5′-GATTTAGTTATTCTTGTTG-3’	48 °C
	ND1_R	5′-CCAACAAAAACACATCTAAC-3’	
*cox*1	COI_F1	5′-GGTGATTATAATGTTTAATG-3’	53 °C
	COI_R1	5′-CGTAGGAACCGCAATAAC-3’	
	COI_F2	5′-TATGGTTTATGCTATTTTAAG-3’	53 °C
	COI_R2	5′-GGCACTACACAACGATTATC-3’	
ITS	ITS_F	5′-GTAGGTGAACCTGCGGAAGGATCATT-3’	55 °C
	ITS_S	5′-TTAGTTTCTTTTCCTCCGCT-3’	

PCR products of *cox*1 were directly sequenced. Since the products of *nad*1 and ITS could not be sequenced due to the heterogeneity caused by PCR incorporated error in the regions of poly-adenine or thymine in *nad*1 or within-individual heterogeneity in ITS, they were instead harvested from the gel over mini-spin columns (Axygen; Union, USA). The purified PCR products were ligated into pGEM®-T Easy vectors with the LigaFast ligation system (Promega; Shanghai, P.R. China). The plasmid vector with the target fragment was transformed into JM109 or DH5α *Escherichia coli*, according to the manufacturer’s instructions. Positive clones were then subjected to sequencing using the dideoxynucleotide termination method. One clone of the *nad*1 gene and three clones of the ITS gene from each specimen were selected for sequencing. All fragments were determined by double-directional sequencing.

### Data from GenBank

Previous studies identified 13 unique clades of the *cox*1 gene of *A. cantonensis* [24]. The typical sequences available in GenBank were utilized to infer the phylogeny. The access numbers are indicated in Fig. 3. The complete *cox*1 sequence of *A. malaysiensis* (KT947979), *A. costaricensis* (GQ398122), *A. vasorum* (JX268542), *Metastrongylus salmi* (GQ888715), *M. pudendotectus* (GQ888714) and *Aelurostrongylus abstrusus* (JX519458) were used as outgroups.

### Data analysis of mitochondrial genes

The sequences of each target gene were aligned using ClustalX version 2.0 [25] and trimmed using BioEdit version 7. The truncated sequences were then presented to DnaSP version 5 [26] to collapse into unique haplotypes for subsequent phylogenetic analysis. Polymorphic sites and DNA polymorphism were analysed by DnaSP version 5. The haplotype diversity (Hd), and nucleotide diversity (Pi) were estimated.

Before reconstructing the phylogenetic tree, the best-fitting nucleotide substitution model was determined by jModeltest version 0.1.1 [27]. Bayesian inference was conducted in MrBayes version 3.1 [28] until the average standard deviation of split frequencies were below 0.01, and the potential scale reduction factor was reasonably close to 1.0 for all parameters. Neighbour-joining and maximum parsimony estimates were performed in Mega version 5.05 [29] with bootstrap testing (1000 replicates). The Bayesian consensus trees were summarised and visualised by Mesquite version 2.75 [30]. The geographical distribution of the clades of the *cox*1 gene was mapped with the geographical information system ArcInfo 9.2.

### Data analysis of ITS

The variable sites of mitochondrial and ITS genes were plotted to compare their validity for phylogeny. At each variable site, the parsimony-informative and -uninformative sites were distinguished, and the proportion of nucleotide substitutions across all sequences was calculated.

A few microsatellites were identified during the initial alignment. Three microsatellites, which showed considerable variation in the number of repeats, were extracted for further analysis. The types for each microsatellite were categorised and the different microsatellite combinations were counted. The distribution of these combinations among phylogenetic clades, determined by mitochondrial genes, was compared.

## Results

### Variation of mitochondrial genes

**Fig. 2:**
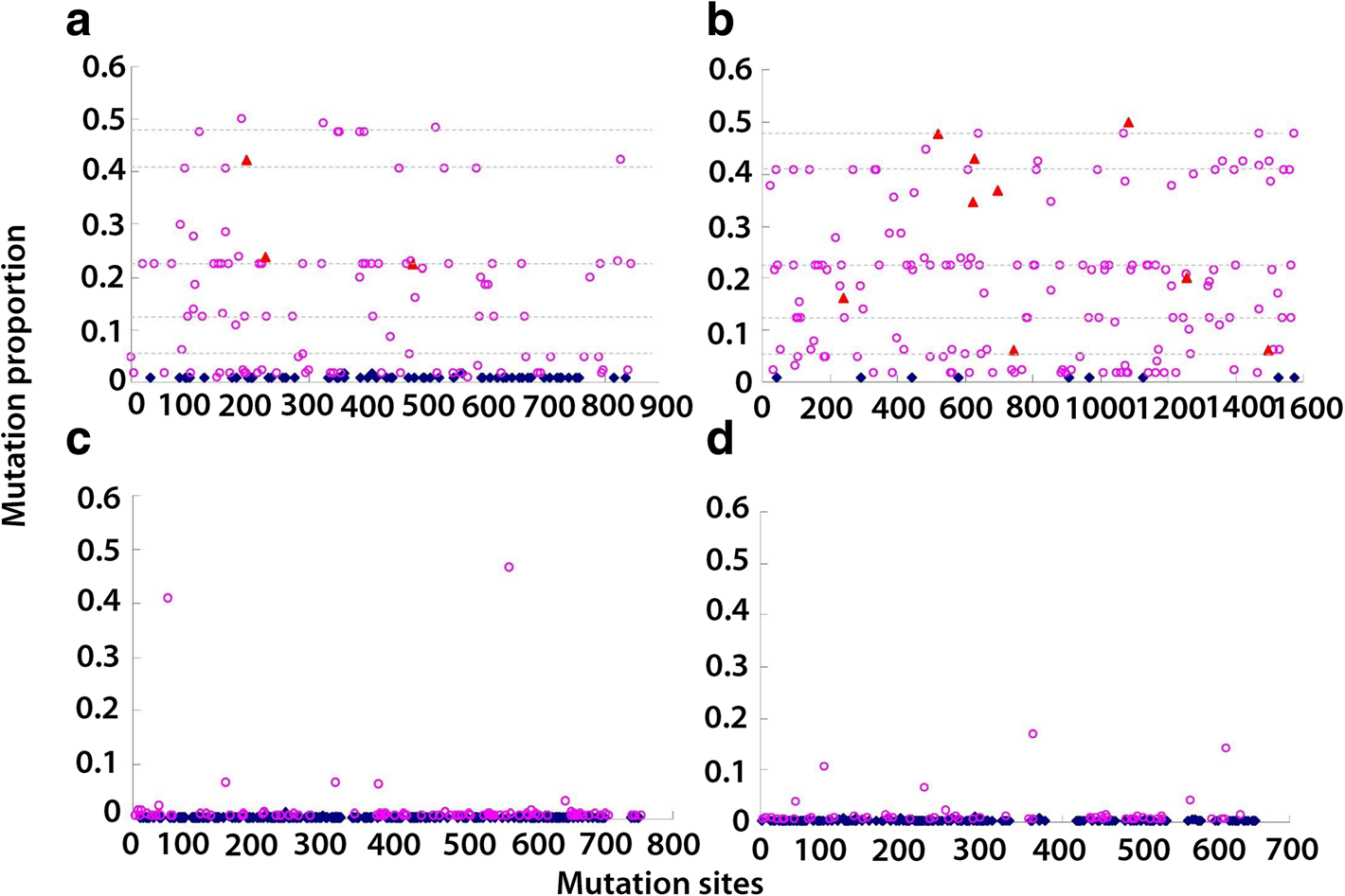
Plots of variable sites by gens; *nad*1 (**a**), *cox*1 (**b**), ITS1 (**c**) and ITS2 (**d**). Three types of variations are shown (*red diamond*, compound parsimony-informative; *pink circle*, simple parsimony-informative; *blue square*, singleton). A compound parsimony-informative site refers to a position where three or four nucleotide types occur simultaneously more than twice in an alignment. The *dot lines* in (**a**) and (**b**) denote parsimony-informative sites with similar mutation proportions

The *nad*1 and *cox*1 genes of a total of 130 *A. cantonensis* specimens from 32 collection sites, representing the whole known endemic area of angiostrongyliasis in P.R. China, were successfully sequenced. Considerable variation was observed both in the sequences of *nad*1 and *cox*1. The number of haplotypes was 39 for *cox*1 (Hd: 0.8114; Pi: 0.0284) and 75 for *nad*1 (Hd: 0.9260; Pi: 0.0314), respectively. Among the 843 sites of the complete *nad*1 gene and 1577 sites of the complete *cox*1 gene, 171 variable sites were identified in each gene. Parsimony-informative sites accounted for the major proportion, i.e. 60.2% in *nad*1 (103/171) and 94.7% in *cox*1 (162/171). The latter is remarkably higher than the former (Fig. 2a and b). However, a considerable proportion of singlets in *nad*1 was observed. It was probably caused by the sequencing strategy, i.e. clone sequencing; PCR incorporation errors got manifested when sequencing clones. Compound parsimony-informative sites, where three or four nucleotide types occurred simultaneously more than twice, were also noted at a frequency of 9 in *cox*1 versus 3 in *nad*1. Additionally, many variable sites showed a similar proportion of nucleotide substitutions, denoted by the dot line in Fig. 2a and b, which indicated a high validity of the gene in distinguishing the clades.

The proteins cytochrome oxidase subunit I (COI) and NADH dehydrogenase subunit 1 (NADH1) were inferred to consist of 525 and 281 amino acids, respectively. Sixteen and 17 variable sites were identified in the presumed protein COI and NADH1, respectively. The corresponding synonymous variations are 18 and 73. Of note, a few variation sites in the protein sequence contain more than two amino acids, which resulted from mutations in a different codon position.

### Phylogeny based on *cox*1 and mapping of clades

**Fig. 3:**
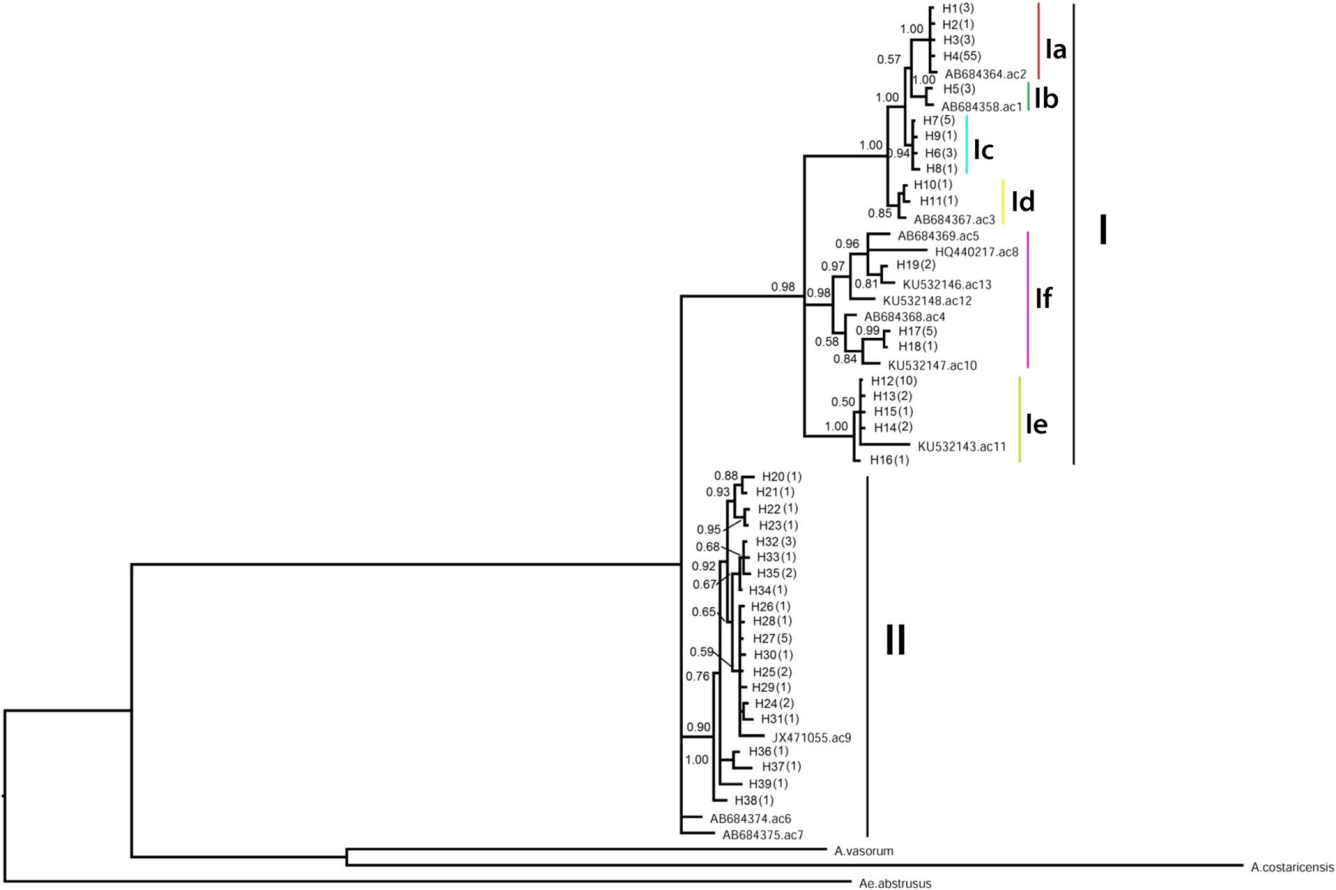
Phylogenetic tree inferred by complete *cox*1 sequences. The posterior probability of Bayesian inference is marked at the branches. The number in brackets after the taxon name is the frequency of the haplotype. The *letters* with the *vertical bar* denote the clades. I and II denote the major groups

In order to avoid false inference, the *nad*1 data were excluded from the construction of the phylogeny. The tree inferred based on the complete sequence of *cox*1 produced in the present study showed two distinct groups, namely I and II (Fig. 3). The group I can be further distinguished into six clades. Almost half of the specimens (47.7%) fell into clade Ia and 22.3% into group II. The haplotypes from clades Ib and Id were limited to single collection sites (Fig. 1). All the 13 known clades of *A. cantonensis* identified by previous studies [14, 24] fell into the clades or groups identified in the present study.

### Variation of ITS

Overall, 357 sequences containing complete ITS1, 5.8S rRNA and ITS2 were obtained from the 130 *A. cantonensis* specimens. According to the structure of ribosomal RNAs of *Caenorhabditis elegans* and *Metastrongylus* [31, 32], the entire ITS1 and ITS2 genes were determined. The length of ITS1 ranged from 708 bp to 743 bp and that of ITS2 from 606 bp to 654 bp. The difference in length within genes mainly resulted from the variable number of repeats in the microsatellites. However, deletion mutations of long fragments (7 bp in six sequences, 9 bp, 22 bp and 40 bp in a single sequence, respectively) outside the microsatellites were also observed in ITS2.

A total of 300 variable sites were identified in ITS1 among 756 aligned sites, and 257 in ITS2 among 654 aligned sites. In contrast to mitochondrial genes, the proportion of parsimony-informative sites was only 32.7% in ITS1 (98/300) and 30.0% in ITS2 (77/257). In addition, there were only 13 and 16 parsimony-informative sites where the proportion of nucleotide substitution across all obtained sequences was higher than 1% in ITS1 and ITS2, respectively. It was significantly lower than those in mitochondrial genes (Fig. 2c and d).

Within-individual heterogeneity in the ITS was also noted. There were 90 specimens for which three complete ITS sequences from each individual specimen were obtained. Slightly less than a third of the specimens (30.0%) showed three distinct haplotypes, determined by 29 parsimony-informative sites, whereas only 15.5% were homogeneous.

### Phylogeny based on ITS

**Fig. 4:**
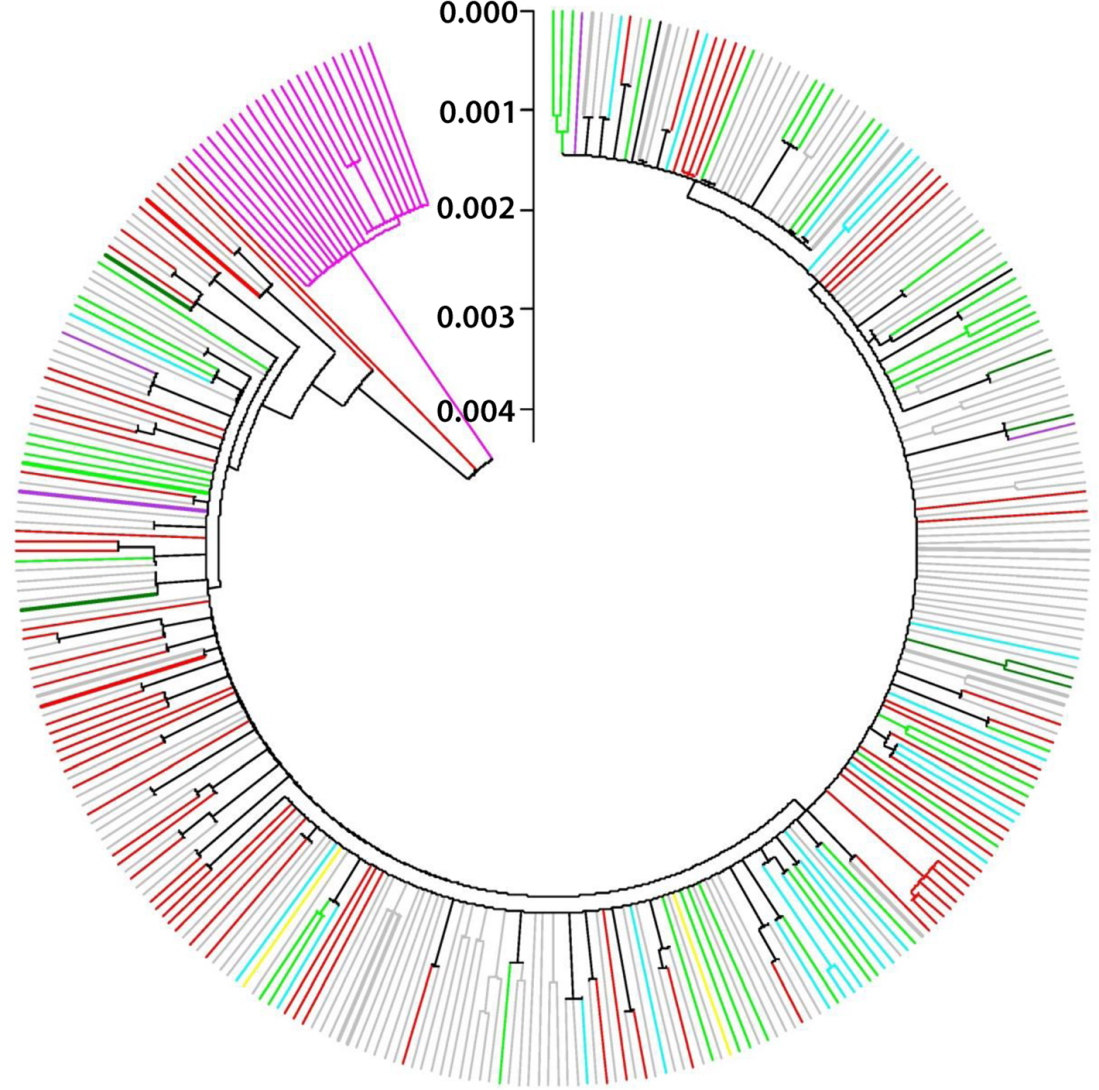
Phylogenetic tree inferred by the complete ITS1, 5.8S and ITS2 sequence excluding the three length-variable microsatellites. The neighbour-joining method with the TrN + G model was used. Taxon name is hidden and the *colors* correspond to the clades in Fig. 3. The *bold branches* indicate that the frequency is more than one

**Fig. 5:**
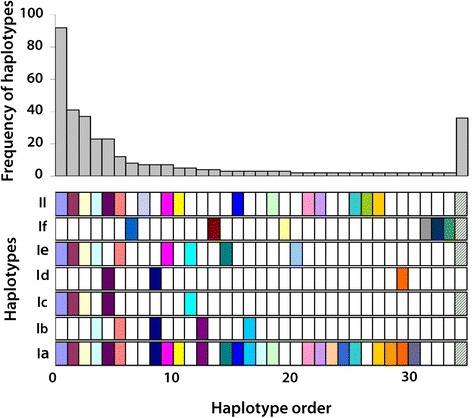
Distribution of haplotypes determined by ITS among the clades inferred by *cox*1 genes. The *top chart* is the frequency distribution of haplotypes, and the *bottom bar chart* indicates the distribution among different clades (denoted by *letters*). Each column represents one haplotype. The last column denotes all other haplotypes with a frequency of 1

Bayesian inference and neighbour-joining did not resolve the in-depth topological relationship between each taxon well. The only consensus in the trees was that the clade If inferred based on mitochondrial genes was separated from any other taxa (Fig. 4). An attempt was made to draw inference by 29 parsimony-informative sites where the proportion of nucleotide substitution was higher than 1%. Yet, this approach revealed similar results. Subsequently, the 29 parsimony-informative sites were used to explore the haplotypes. Seventy unique haplotypes were identified and 36 of them occurred with a frequency of 1. The distribution of these haplotypes among the clades inferred by mitochondrial genes is shown in Fig. 5. A striking cross-transmission of haplotypes was observed among all clades, except for clade If. The number of shared haplotypes between each of the two clades increased with the growing pool of sequences.

### Variation of ITS microsatellites

**Fig. 6:**
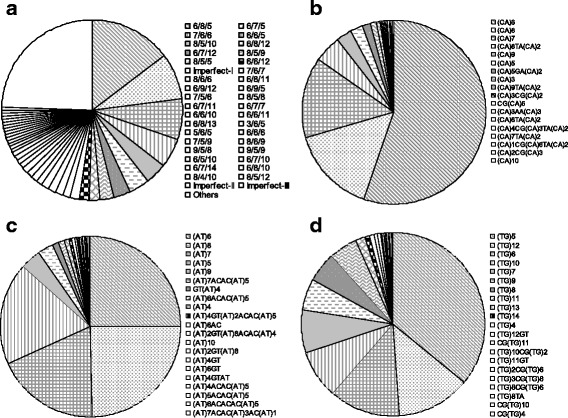
Type of microsatellites; combination of three microsatellites (**a**), MS-5 (**b**), MS-8 (**c**) and MS-12 (**d**). Only 10 types with higher frequencies were denoted using different *filled pies*, with the others indicated by *hollow pies*. In Fig. 6a, the numbers separated by slash denotes the number of repeats in MS-5, 8 and 12, respectively

Twelve microsatellites were observed in the ITS1 and ITS2 sequences (Table 2). Three microsatellites (MS-5, 8 and 12) showed considerable variation in the number of repeat units, which was the major cause of the low success of direct sequencing of PCR products. The three microsatellites showed perfect repeats as well as imperfect repeats (Fig. 6). A total of 18 repeat types were found in MS-5. The other two microsatellites had 21 types, respectively. The diversity significantly increased when the three microsatellites were concatenated; there were 126 combinations among all the 357 ITS sequences. However, the majority of them (86) occurred with a frequency of 1.

**Table 2:** Microsatellites (MS) identified from the alignment of concatenated ITS1 and ITS2

MS	Position	Length	Repeats	Sequence
1	73	3	3	CGTCGTCGT
2	100	4	3	GGTGGGTGGGTG
3	118	3	3	TGATGATGA
4	255	2	3	ACACAC
5	329	2	Variable	(CA)n
6	401	2	3	TGTGTG
7	478	2	4	TGTGTGTG
8	727	2	Variable	(AT)n
9	1178	4	4	ATCGATCGATCGATCG
10	1282	2	3	GTGTGT
11	1446	2	3	ACACAC
12	1468	2	Variable	(TG)n

It was also noted that some imperfect repeats could represent the special clades inferred by mitochondrial gene analysis. For example, (CA)_n_TA(CA)_m_ in MS-5 and (AT)_n_ACAC(AT)_m_ in MS-8 only occurred in clade If. Furthermore, almost all the specimens in this clade showed the two imperfect repeats simultaneously.

**Fig. 7:**
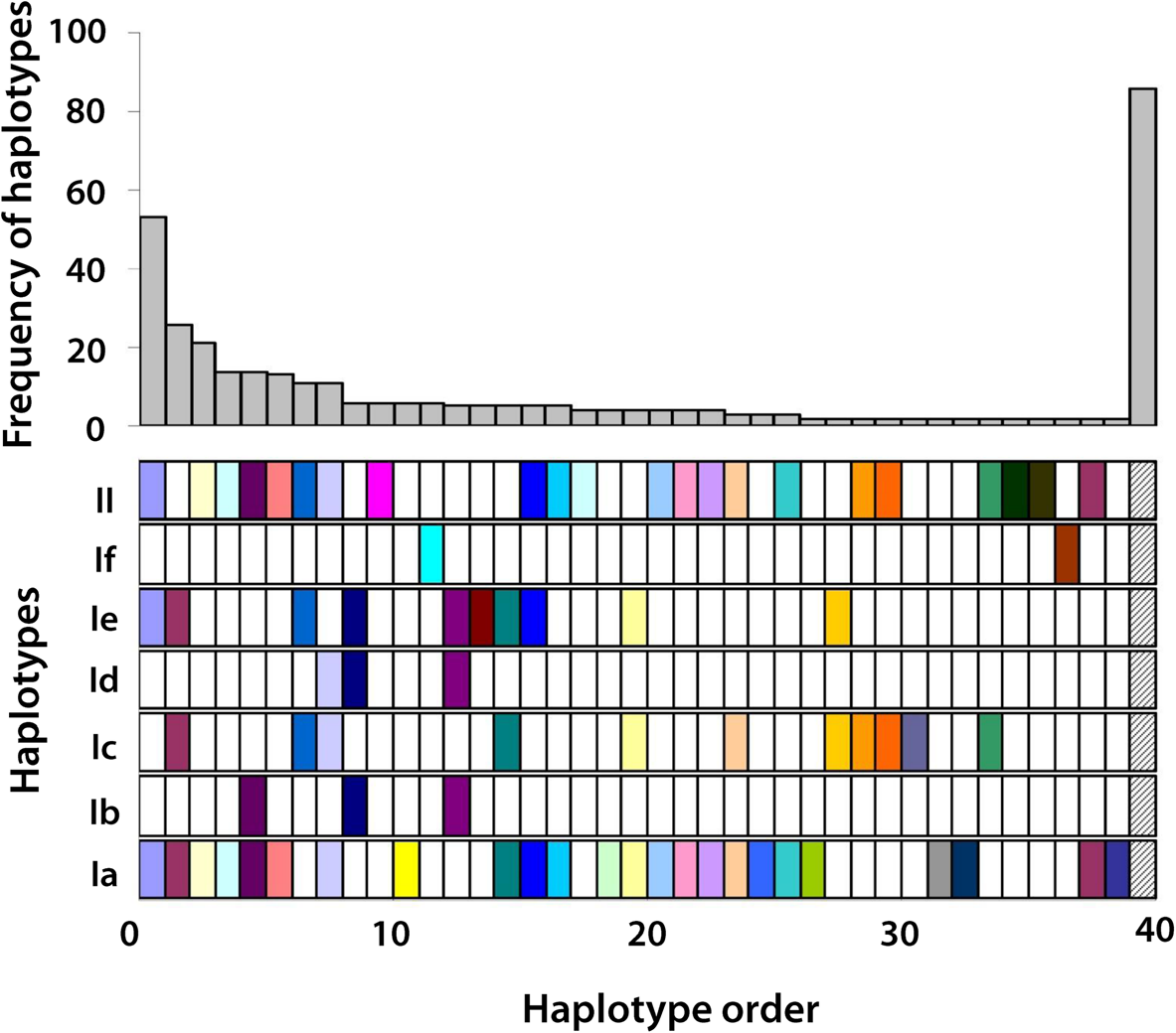
Distribution of haplotypes determined by microsatellites among the clades inferred by mitochondrial genes. Only MS-5, 8 and 12 were included in the analysis. For other labels, the reader is referred to Fig. 5

With regard to microsatellites, we also explored the distribution of these combinations among the clades inferred from mitochondrial genes (Fig. 7). Similarly, a notable cross-transmission occurred across all clades except for clade If, although the pattern was different from that derived from ITS. We also noted that clades Ia and II shared much more types than the other clades.

We observed considerable individual heterogeneity of microsatellite types. Two-third of the 90 samples, for which three complete ITS sequences were obtained, showed three distinct types, whereas only four samples (4.4%) were homogeneous. The proportion (66.7%) of individuals which had three distinct microsatellite types was much higher than that (30.0%) of individuals which had three distinct ITS haplotypes. The conserved estimation in ITS haplotypes only using 29 parsimony-informative sites might explain this lower proportion.

## Discussion

The distribution of *A. cantonensis* in P.R. China is currently restricted to eight southern provinces [21]. As suggested by recent modelling work, it could change due to global warming, the further spread of invasive snail species and changing transmission dynamics [33]. We identified seven distinct clades based on the analysis of mitochondrial genes, which indicates a high diversity of *A. cantonensis* in its current presumptive home range. The major clades identified are Ia and II. Although *A. malaysiensis* was reported to occur in close proximity to the border with Vietnam [34], our results show that *A. cantonensis* is probably the only species endemic in mainland P.R. China. We further excluded the possibility of group II being a separate species, since there was a lack of essential differences in ITS sequences between this group and other clades. In addition, the wide cross-transmission of ITS genotypes and microsatellites implies the absence of any reproductive isolation. Furthermore, the genetic distance between group II and *A. malaysiensis* is long (results not shown due to a long-branch attraction). Instead, group II was much closer to the other clades of *A. cantonensis*.

Although clade If was found to be more closely related to clade Ia (the most common haplotype in P.R. China) than group II, nuclear genes indicated that clade If is a distinctive group. Indeed, there is no cross-transmission of haplotypes as inferred by ITS and microsatellites between clade If and any of the other clades. We speculate that geographical isolation is the root cause of this observation. The *A. cantonensis* specimens obtained from Yunnan Province fell into clade If, along with samples from Thailand. Thus, the samples collected from areas near the border to Myanmar might belong to the same transmission region as the Thai isolates. In contrast, we infer that group II probably was introduced into the mainland of P.R. China. Hybridization might play an important role in the nuclear similarity between group II and other presumptive native clades. However, the fate of original nuclear genetic characters of group II and/or native clades remains to be investigated.

We noted that the proportion of singleton variable sites is strikingly variable between nuclear genes and mitochondrial genes, and even among mitochondrial genes. First, there is a significantly higher proportion of singleton variable sites in *nad*1 (39.8%) compared to *cox*1 (5.3%). Second, singleton variable sites account for 67.3% of variable sites in ITS1 and 70.0% in ITS2, which is notably higher than in mitochondrial genes. Sequencing methods might explain the difference between *cox*1 and *nad*1, and further between mitochondrial genes and ITS sequences. Sequencing following cloning is sensitive to PCR-induced artifacts, and hence, might falsely increase the apparent diversity [35, 36]. Therefore, direct sequencing of PCR product is recommended for future studies.

We found striking intragenomic or within-individual heterogeneity in both ITS sequences and microsatellites, which conflict with concerted evolution [37] but is consistent with previous studies [38, 39]. Although we reduced false diversity induced by cloning sequencing as mentioned above by excluding the sites where the proportion of nucleotide substitution was less than 1%, our conserved estimate of within-individual heterogeneity using 29 parsimony-informative sites is still notable. This finding demonstrates that ITS is not a useful genetic marker for population genetic studies of *A. cantonensis*.

The length of ITS region in different nematode species shows considerable variation. For example, the ITS region of some representative species from Rhabditid and Cephalobid orders is between 275 bp and 875 bp long [40]. Our findings and previous studies indicate that the ITS length of the genus *Angiostrongylus* may be much longer than that of other nematodes [18, 41]. Indeed, the combination of ITS1 and ITS2 can be as long as 1377 bp, as described here. Like other members of the genus *Angiostrongylus* [42], *A. cantonensis* also shows microsatellites in the ITS regions and some of them are strikingly variable in the number of repeats, which is a challenge for direct sequencing of PCR products. Our findings suggest that the primer designation for ITS regions should avoid the microsatellites to potentially increase the success of direct sequencing of PCR products, which in turn will lower the number of artifacts induced by PCR.

## Conclusions

We conclude that *A. cantonensis* is the only *Angiostrongylus* species in the study area. Our results show a high diversity of *A. cantonensis* in mitochondrial genes, which is helpful to elucidate the global spread from a molecular perspective. We observed intragenomic heterogeneity in ITS. Hence, ITS appears not to be an appropriate marker for genotyping of *A. cantonensis*. The sequencing strategy can considerably impact the haplotype diversity, and hence, clone sequencing cannot be recommended.

## Additional file

Additional file 1: Multilingual abstracts in the six official working languages of the United Nations. (PDF 663 kb)

## Abbreviations

CNS: Central nervous system
*cox*1: The gene of cytochrome c oxidase subunit I
FTA: Flinders Technology Associates (card)
Hd: Haplotype diversity
ITS: Internal transcribed spacer
MS: Microsatellites
*nad*1: The gene of nicotinamide adenine dinucleotide dehydrogenase subunit 1
PCR: Polymerase chain reaction
Pi: Nucleotide diversity
SD: Sprague-Dawley (rat)
