# Supplementary material for: The genetic variation of Angiostrongylus cantonensis in the People’s Republic of China
Source: Infect Dis Poverty. 2017 Sep 1;6:125. doi: 10.1186/s40249-017-0341-z (PMC5579933; doi:10.1186/s40249-017-0341-z)
Supplement: Additional file 1: — Multilingual abstracts in the six official working languages of the United Nations. (PDF 663 kb) [file 40249_2017_341_MOESM1_ESM.pdf]

Translation of the abstract into the six official working languages of the United Nations

الاختلاف الجيني للدودة الأسطوانية الكنتونية في جمهورية الصين الشعبية

شان لف، يي تشانج، بيتر شتاينمان، يورج أوتزينجر، شياو نونج تشو

ملخص

خلفية: جمهورية الصين الشعبية هي المأوى الافتراضي لدودة الرئة في الفئران Angiostrongylus cantonensis، وهي عامل مسبب رئيسي لالتهاب السحايا اليوزيني في الإنسان. نقدم دراسة عن الاختلاف الجيني لدودة A. cantonensis في جمهورية الصين الشعبية. هدفنا هو تعميق المعرفة الحالية المتعلقة بأصلها وانتشارها على المستوى العالمي من منظور الجزيئي.

أساليب: تم تجميع ديدان A. cantonensisالبالغة في مسح وطني وتحديدها في المقام الأول على أساس المعايير المورفولوجية. تم استخدام تفاعل البوليميراز المتسلسل (PCR) لتضخيم تسلسل الحمض النووي المستهدف (1cox، 1nad والفاصلات الداخلية للقطع المنسوخة ITS). تم تقديم منتج جمهورية الصين الشعبية من 1cox مباشرة إلى التسلسل، في حين تم استخدام تسلسل استنساخ ل 1nad و الفاصلات الداخلية للقطع المنسو تم التحقق من هوية العينات عن طريق مقارنة التسلسلات مع تلك المقبولة من عيناتA. cantonensis. تم تحليل تركيبة محددة من البدائل في كل جين، وتمت مقارنة الأنماط الجينية على أساس كامل جينات 1cox، 1nad والفاصلات الداخلية للقطع المنسوخة.

النتائج: لقد ميزنا جينات الميتوكوندريا الكاملة 1cox و 1nad ضمن 130 عينة وحصل على 357 تسلسل نووي يحتوي على اثنين من الفواصل الداخلية مكتوبة كاملة (ITS1 و ITS2) والرنا الريباسي S5.8 من نفس العينات. تم تأكيد أن جميع العينات الوراثية من A. cantonensis. تم تحديد مجموعتين رئيسيتين (أي الأولى والثانية) وفقا لنشوء متواليات1cox . المجموعة الأولى يمكن تصنيفها إلى ستة فصائل متميزة. تقريبا نصف العينات (47.7٪) تنتمي إلى الفصيلة A-I و 22.3٪ للمجموعة الثانية. وزعت الأولى على نطاق واسع في جميع أنحاء منطقة الدراسة. ولوحظ عدد متغير من الوحدات المتكررة في ثلاثة سواتل صغرية، مما أدى إلى تباين كبير في الطول في الفاصلات الداخلية للقطع المنسوخة. تم العثور على اختلاف داخل الجينوم في تسلسلات الفاصلات الداخلية للقطع المنسوخة في نسبة كبيرة من العينات. حدث اختلاف ملحوظ في التنميط الجيني بين الحمض النووي للميتوكوندريا وتسلسلات الفاصلات الداخلية للقطع المنسوخة.

الاستنتاجات: تثبت نتائجنا أنA. cantonensis هي النوع الوحيد من فصيلة الديدان الرئوية في الفئران في جمهورية الصين الشعبية ويظهر بها تنوع وراثي واضح. نتائج التنوع والتنميط الجيني لـ A. cantonensis يمكن أن تتأثر باستراتيجية التسلسل والعلامات البيولوجية. على الرغم من أن الفاصلات الداخلية للقطع المنسوخة قد تكون علامة قيمة لتحديد بين الأنواع، فإنها ليست مناسبة لدراسة الاختلاف بين أنواعA. cantonensis وذلك بسبب الاختلاف الواضح داخل الجينوم والتحديات الحالية للتسلسل المباشر.

Translated from English version into Arabic by Mahmoud Sami, through

TRANSLATORS WITHOUT BORDERS

中国广州管圆线虫的遗传多样性

吕山, 张仪, Peter Steinmann, Jürg Utzinger, 周晓农

摘要

引言：中国是人类嗜酸性脑膜脑炎病原体广州管圆线虫的起源地。本研究将展示中国的广州管圆线虫遗传变异，目的在于从分子角度加深对广州管圆线虫起源与全球扩散的理解。

方法: 广州管圆线虫成虫收集自全国调查，并通过形态学进行初步鉴定。利用 PCR 技术扩增目的基因序列（*cox*1，*nad*1 和 ITS）。*cox*1 基因的 PCR 扩增产物直接测序，而 *nad*1 和 ITS 基因的 PCR 扩增产物则采用克隆测序的方法。标本虫种的鉴定结果通过与已知广州管圆线虫的序列比对进行确认。逐一分析每个基因序列的碱基替代组成，并比较基于 *cox*1，*nad*1 和 ITS 基因全长序列获得的基因型。

结果: 我们从 130 个标本中获取了 *cox*1 和 *nad*1 基因全长序列，同时获得了包含 2 个完整的间插序列（ITS1 和 ITS2）和 1 个 5.8s 核糖体 RNA 序列的 357 个核基因序列。所有的序列均被确认为广州管圆线虫的基因序列。根据 *cox*1 序列构建的进化树将这些标本区分为 2 个群（即 I 和 II）。第 I 群又进一步分为 6 个独立的分枝。几乎一半的标本（47.7%）属于分枝 Ia，22.3%属于第 II 群。前者广泛分布于该研究区域。3 个微卫星序列展示了重复子的变异多态性，进而导致 ITS 长度的变异。ITS 序列在多数样本中都展示了基因组内变异。线粒体基因和 ITS 所建立的基因型有显著的差异。

结论: 我们的研究结果显示广州管圆线虫是中国唯一鼠肺线虫虫种，并展示了高度多样性。广州管圆线虫的多样性和基因分型结果可受测序策略和生物标记物的影响。尽管 ITS 可以作为种间的鉴定标记物，但因其基因组内高度变异和直接测序的困难而不适合作为研究广州管圆线虫种内变异的标记物。

Translated from English version into Chinese by Shan Lv

Variations génétiques d'*Angiostrongylus cantonensis* en République populaire de Chine

Shan Lv, Yi Zhang, Peter Steinmann, Jürg Utzinger, Xiao-Nong Zhou

Résumé

Contexte: La République populaire de Chine (RPC) est probablement le territoire d'origine du nématode *Angiostrongylus cantonensis*, parasite du rat et agent majeur de méningites à éosinophiles chez l'homme. Nous présentons ici une étude des variations génétiques d'*A. cantonensis* en République populaire de Chine. Notre but est d'approfondir les connaissances actuelles concernant son origine et sa propagation mondiale, à l'aide des méthodes moléculaires.

Méthodes: Des spécimens adultes d'*A. cantonensis* ont été recueillis au cours d'une campagne de collecte nationale et identifiés, pour commencer, sur la base de critères morphologiques. La réaction en chaîne à la polymérase (RCP) a été employée pour amplifier les séquences d'ADN cibles (*cox*1, *nad*1 et ITS). Le produit de RCP de *cox*1 a été séquencé directement, celui de *nad*1 et ITS après clonage. L'identité des échantillons a été vérifiée en comparant les séquences à celles de spécimens d'*A. cantonensis* reconnus. La composition spécifique des substitutions de chaque gène a été analysée et les génotypes ont été comparés sur la base des gènes *cox*1, *nad*1 et ITS complets.

Résultats: Nous avons caractérisé entièrement les gènes mitochondriaux*cox*1 et *nad* de 130 spécimens et obtenu 357 séquences contenant deux espaceurs transcrits internes complets (ITS1 et ITS2) et l'ARNr 5,8S des mêmes échantillons. L'identification de tous les spécimens comme *A. cantonensis* a été confirmée par la génétique Deux grands groupes (I et II) ont été identifiés, selon la phylogénie des séquences *cox*1. Le groupe I a été subdivisé en six clades distincts. Près de la moitié des échantillons (47,7 %) appartiennent au clade Ia et 22,3 % au groupe II. Le premier clade était largement distribué dans toute la région de l'étude. Un nombre variable d'unités répétées a été observé dans les trois microsatellites, avec pour résultat une variation

considérable de la longueur d’ITS. Une variabilité intragénomique des séquences ITS a été observée dans une grande proportion des échantillons. Des différences frappantes de génotype ont été découvertes entre l’ADN mitochondrial et ITS.

Conclusions: Nos résultats démontrent qu’*A. cantonensis* est la seule espèce du genre parasite du rat présente en RPC et que sa diversité génétique est considérable. Les résultats de diversité et de génotypage d’*A. cantonensis* peuvent dépendre de la stratégie de séquençage et du biomarqueur. Bien que la séquence ITS soit un marqueur précieux pour la discrimination interspécifique, elle ne convient pas pour étudier les variations intraspécifiques d’*A. cantonensis*, en raison de sa forte variabilité intragénomique et des difficultés actuelles du séquençage direct.

Translated from English version into French by Suzanne Assenat, through

Генетическая изменчивость *Angiostrongylus cantonensis* в Китайской Народной Республике

Шань Лв Йи Чжан, Питер Штейнман, Юрг Уцзингер, Сяо Нонг Чжоу

Реферат

Справочная информация: Китайская Народная Республика (КНР) предположительно считается очагом распространения крысиного лёгочного червя*Angiostrongylus cantonensis*, основного этиологического агента, являющегося возбудителем эозинофильного менингита. Предметом данного исследования является изучение генетической вариации *a. cantonensis* в Китае. Цель работы заключается в углублении уже существующих знаний относительно как происхождения крысиного лёгочного червя, так и изучение его повсеместного распространения с молекулярной точки зрения.

Методы: Образцы взрослого *лёгочного червя* были получены в ходе национального обследования, идентификация произведена главным образом по морфологическим признакам. С целью амплифации последовательностей ДНК-мишени (*cox*1, *nad*1 и ITS) была задействована полимеразная цепная реакция (ПЦР). В то время как продукт ПЦР с *cox*1 был напрямую задействован в последовательности, в случае с*nad*1 и ITS было использовано последовательное клонирование. Информация о предоставивших образцы была проверена путем сравнения последовательностей принятых к анализу образцов *A. cantonensis*. Наряду с анализом конкретного состава замещений по каждому гену было произведено сопоставление генотипов на основе полного комплекта геномов *cox*1, *nad*1 и ITS.

Результаты: В ходе анализа 130 образцов была произведена характеризация полных митохондриальных геномов *cox*1 и *nad*1, а также получены 357 ядерных последовательностей, содержащих два полных внутренних спейсера ДНК (ITS1 и ITS2) и 5.8S рРНК с тех же образцов. Принадлежность к *a. cantonensis*была генетически подтверждена по всем образцам. В соответствии филогенией последовательностей *cox*1 были выявлены две основные группы (I и II). В дальнейшем группа I может

быть разбита на шесть отдельных клад. Почти половина образцов (47,7%) принадлежит кладе Ia, тогда как 22,3% относятся к группе II. В первом случае распределение было произведено по всему региону, подвергшемуся исследованию. Было отмечено присутствие переменного числа повторных единиц в трех микроспутниках, что привело к удлиннению изменчивости ITS. В значительной пропорции образцов была обнаружена внутрегеномная изменчивость последовательностей ITS. Поразительное различие генотипирования произошло между митохондриальной ДНК и ITS.
Выводы: Результаты исследования указывают на то, что *A. cantonensis* является единственным крысиным лёгочным червем на территории Китайской Народной Республики, демонстрирующим высокий уровень генетический изменчивости. Результаты изменчивости и генотипирования *A. cantonensis* могут быть подвержены влиянию как стратегии секвенсирования, так и биомаркерам. Несмотря на то, что ITS способен оказаться ценным маркером внутреннего специфического определения, указанный показатель не подходит для изучения интраспецифической изменчивости*A. cantonensis* вследствие его высокой интрагенной изменчивости, а также существующих трудностей с прямым секвенсированием.

Translated from English version into Russian by Liudmila Tomanek (nee Volynets) , through

TRANSLATORS
WITHOUT BORDERS

La variación genética de *Angiostrongylus cantonensis* en la República Popular China.

Shan Lv, Yi Zhang, Peter Steinmann, Jürg Utzinger, Xiao-Nong Zhou

Resumen

Antecedentes: la República Popular China (China) es el presunto área de acción de *Angiostrongylus cantonensis*, un importante agente causal de la meningitis eosinofílica humana. Presentamos un estudio de la variación genética de *A. cantonensis* en China. Nuestro objetivo es profundizar en los conocimientos actuales sobre su origen y propagación mundial desde una perspectiva molecular.
Método: Se recopilaron especímenes adultos de *A. cantonesis*en un estudio nacional y se identificaron principalmente por sus características morfológicas. Se empleó la reacción en cadena a la polimerasa (RCP) para aumentar las secuencias meta de ADN (*cox*1, *nad*1 e ITS). El resultado de la RCP de *cox*1 se sometió directamente a secuenciación, si bien la secuencia de clonado fue empleada para *nad*1 e ITS. La identidad de las muestras se verificó mediante la comparación de las secuencias con las de los especímenes de *A. cantonensis* aceptados. Se analizó la composición específica de las sustituciones en cada gen, y se compararon los genotipos en función de los genes completos de *cox*1, *nad*1 e ITS.
Resultados: Hemos caracterizado los genes mitocondriales completos *cox*1 y *nad*1 de 130 especímenes y obtuvimos 357 secuencias nucleares que contenían dos espacios intergénicos completos (ITS1 e ITS2) y 5.8S ARN de las mismas muestras. Se confirmó genéticamente que todos los especímenes eran *A. cantonensis*. Se

identificaron dos grandes grupos (I y II) según la filogenia de las secuencias de *cox*1. El grupo I podría subcategorizarse en seis distintos subtipos. Casi la mitad de las muestras (47,7%) pertenecen al subtipo Ia y un 22.3% al subtipo II. Este último subtipo se distribuyó ampliamente en el área de estudio. Se observó un número variable de unidades de repetición en tres microsatélites, lo que derivó en una variación de longitud considerable en ITS. Se encontró una variación intragenómica de las secuencias de ITS en gran parte de las muestras. Hubo una sorprendente diferencia de genotipado entre el ADN mitocóndrico e ITS.
Conclusiones: Nuestros resultados indican que *A. cantonesis* es la única especie de estrongílidos de China y muestra una alta diversidad genética. Los resultados de la diversidad y el genotipado de *A. cantonensis* pueden depender de la estrategia de biomarcado y secuenciación. Aunque ITS puede ser un valioso marcador para la identificación interespecífica, no es adecuado para el estudio de la variación intraespecífica de *A. cantonensis* debido a su alta variación intragenómica y a los desafíos actuales de la secuenciación directa.

Translated from English version into Spanish by cavigar, through

TRANSLATORS
WITHOUT BORDERS
